# Coexistence of aortic valve stenosis and cardiac amyloidosis: echocardiographic and clinical significance

**DOI:** 10.1186/s12947-019-0182-y

**Published:** 2019-12-26

**Authors:** Gergely Peskó, Zsigmond Jenei, Gergely Varga, Astrid Apor, Hajnalka Vágó, Sándor Czibor, Zoltán Prohászka, Tamás Masszi, Zoltán Pozsonyi

**Affiliations:** 1 3rd Department of Internal Medicine, Semmelweis University, Kútvölgyi 4, Budapest, 1125 Hungary; 2 Heart and Vascular Center, Semmelweis University, Városmajor 68, Budapest, 1122 Hungary; 3 Department of Nuclear Medicine, Semmelweis University, Üllői 78, Budapest, 1082 Hungary

**Keywords:** Cardiac amyloidosis, Aortic valve stenosis, Dobutamine stress echo

## Abstract

**Background:**

Left ventricular hypertrophy and diastolic dysfunction are common echocardiographic features of both aortic valve stenosis (AS) and cardiac amyloidosis (CA). These two different entities therefore may mask each other. From recent years, there is a growing body of evidence about the relatively high incidence of wild-type transthyretin (wtTTR) amyloidosis in AS, but there are scarce data on the prevalence of AS in CA, particularly in AL-type amyloidosis. The echocardiographic approach to these patients is not obvious, and not evidence based. We aimed to study the prevalence, severity, and type of AS in patients with CA and also to evaluate the potential of echocardiography in the diagnostic process.

**Methods:**

Between January 2009 and January 2019, we retrospectively analyzed the clinical and echocardiographic data, and the echocardiographic work up of 55 consecutive CA patients.

**Results:**

80% of our CA patients had AL amyloidosis. We identified 5 patients (9%) with moderate to severe AS: two with moderate AS and three with low-flow, low-grade AS (LFLG AS). Further analysis of the latter three patients with dobutamine stress echocardiography revealed pseudo-severe LFLG AS in two, and true-severe AS in one patient.

**Conclusion:**

The prevalence of moderate to severe AS is 9% in our population of CA patients, the majority of whom have AL amyloidosis. Dobutamine echocardiography seems to be appropriate for the further characterization of patients with LFLG AS, even with normal ejection fraction.

## Introduction

Aortic valve stenosis (AS) is a common valvular heart disease, which significantly reduces life expectancy when it becomes symptomatic [1]. The prevalence of AS is increasing with age, becoming as high as 12.4 and 3.4% for severe AS in a patient population aged more than 75 years, according to a recent metaanalysis [2]. Cardiac amyloidosis (CA) is generally considered a rare condition caused by protein deposition in the heart. The two most common forms are monoclonal light chain amyloidosis (AL), and transthyretin (TTR) CA [3]. In the former, plasma cell dyscrasia (PCD) results in the production of monoclononal light chains, which infiltrate the heart. In wild-type TTR (wtTTR) amyloidosis seen in elderly patients, the wild type of TTR is responsible for the beta-sheet structure of the protein deposition. Mutant TTR (mTTR) may also infiltrate the heart; this form of the disease becomes symptomatic in the 6th or 7th decades of life.

AS and CA share common echocardiographic features, such as left ventricular hypertrophy, diastolic dysfunction, and – in many cases – elevated left ventricular filling pressure. Echocardiography may not reveal CA in AS, because the typical signs of CA may be readily explained by the AS. In recent years, a few case studies reported the coexistence of wtTTR cardiac amyloidosis and AS, highlighting the diagnostic, as well as the therapeutic challenges of these cases [4, 5]. The studies conducted in AS patients [6–10] to examine the prevalence of TTR amyloidosis in severe AS used diverse diagnostic methods and evaluated different study populations. Some of these studies [6, 7, 9] included more than 100 AS patients. Remarkably, the incidence of TTR CA was as high as 6 to 16% in patients with AS. This question can also be examined the other way around, by determining the prevalence of AS among CA patients. Among TTR CA patients, the prevalence of moderate to severe AS was found to be as high as 27% [11]. However, the prevalence of AS in consecutive CA patients – including those with TTR and AL – has not been examined yet.

We aimed to study the prevalence, severity, and type of aortic valve stenosis in patients with CA. We also aimed to examine the potential role of dobutamine stress echocardiography in the diagnostic work up of patients with CA and low flow-low grade AS (LFLG AS).

## Materials and methods

We performed a systematic review of CA patients treated in our department, between 2009 January and 2019 January, focusing on the presence of AS. We diagnosed AL-type amyloidosis when all the following criteria were met: the presence of monoclonal light chain in the serum and in the urine; the presence of plasma cell dyscrasia in bone marrow biopsy; and positive tissue biopsy confirming light chain amyloid deposition in any organ. Cardiac involvement was diagnosed when cardiac MRI showed late gadolinium enhancement as a typical sign of CA, echocardiography revealed characteristic abnormalities, or the presence of AL amyloid was detected in cardiac tissue by biopsy, performed either during right heart catheterization or at autopsy. Since June 2016, if no monoclonal light chain was present in the serum, we established the diagnosis of TTR CA after a positive Tc-99-pyrophosphate (PYP) isotope scan (Perugini score 2 or 3), specific for the disease [12]. In previous years, positive immunohistology for TTR in tissue biopsy, and the absence of monoclonal light chain in the serum was necessary for diagnosis, along with typical cardiac imaging results. We performed TTR gene sequencing in all TTR cases.

Unless it was contraindicated, we routinely performed CMR (Philips Achieva, 1,5 T, Philips, Amsterdam, The Netherlands) for differential diagnostic purposes, and as a test to confirm CA. Resting echocardiography was performed in accordance with the topical guidelines (Philips iE33, Philips, Amsterdam, The Netherlands) [13–15]. After 2015 for patients with suspicion of CA we performed left ventricular longitudinal strain analysis in order to examine segmental differences, and looked for “apical sparing”. QLab 10.5 cardiovascular ultrasound quantification software was used (Philips, Amsterdam, The Netherlands) for all strain analysis. Following the method of Phelan et his al., [16] the difference of regional left ventricular longitudinal strain (LS) between the apical and other segments of the left ventricle was examined on the bull’s eye image, and calculated with the following equation: Relative apical LS = average apical LS/(average basal LS + average mid LS).

During echocardiography, the suspicion of AS was raised by valve morphology in agreement with the criteria set out in the latest European guideline on valvular heart disease [1]. We performed all the routine echocardiographic measurements for AS. When the indexed aortic valve area was less than 0.6 cm^2^, but low–flow, low-grade AS (LFLG AS) was diagnosed by echography at rest, we performed dobutamine stress testing to differentiate between pseudo- and true-severe AS, regardless of the left ventricular ejection fraction.

Since most of the variables exhibited skewed distributions, the descriptive statistics are presented as medians with interquartile ranges (IQR), or as percentages. The strength of the associations was calculated with the nonparametric Mann-Whitney test or the chi-square test, as appropriate.

## Results

Between January 2009 and January 2019 we performed resting transthoracic echocardiography in 55 patients with cardiac amyloidosis: 44 had AL-, 9 TTR (6 with mTTR and 3 with wtTTR), and one patient had AA amyloidosis. Their median age was 65 years.

Here we summarize the most important therapeutic and mortality data of this patient population: Specific treatment of the AL patients for the plasma cell dyscrasia was driven by hematologists. In most of the cases, a combination of cyclophosphamide-bortezomib and corticosteroid was introduced first, but the whole spectrum of multiple myeloma medication was used, including lenalidomide, thalidomide, daratumumab and melphalan. Doxycylin was used in the last 4 to 5 years. Two patients had autologous bone marrow transplantation.

wtTTR patients did not receive any specific treatment. Four out of the six mTTR patients received 20 mg tafamidis, daily. Generally, our CA patients had no ICD, and none of these 55 patients had heart transplantation.

Calculation of mortality rate is not simple, because the follow up period is no more than nine month in the cases of the latest included patients. We think, the most correct way to describe mortality in this case, is to describe the nine month mortality, which was 23/55 (42%) for the whole CA patient population.

The systematic review identified 5 patients with moderate or severe aortic valve stenosis. Important clinical, biomarker, and echocardiographic characteristics of the patients are shown in Table 1. All 5 patients with AS had symptoms of severe heart failure, had stage III or IV symptoms according to the New York Heart Association’s classification. In three patients, echocardiography was performed for suspected cardiac amyloidosis, as plasma cell dyscrasia was diagnosed before the onset of the signs of heart failure. In the two other cases echo was requested by the clinician, due to symptoms of heart failure. We did not find significant differences in the clinical characteristics of the patients with AS and CA or with CA only; however, this is possibly due to the relatively small sample size. Patients with AS and CA were older, but the *p*-value of the difference was only 0.055.

**Table 1:** Clinical characteristics, serum levels of cardiac biomarkers, and the main echocardiographic parameters of the 55 CA patients, grouped according to the presence or absence of aortic valve stenosis

	Patients without AS (*n* = 50)	Patients with AS (n = 5)	*p*-value
Clinical data			
Age (years)	63.5 (58–73)	69 (68–82)	*p* = 0.055
Male (n, %)	26 (52%)	3 (60%)	*p* = 0.553
wtTTR CA (n, %)	2 (4%)	1 (20%)	*p* = 0.391
mTTR CA (n, %)	6 (12%)	0 (0%)	*p* = 0.485
AL amyloidosis (n, %)	40 (80%)	4 (80%)	*p* = 0.741
AA amyloidosis (n, %)	1 (2%)	0 (0%)	*p* = 0.909
NYHA III-IV stage (n, %)	32 (64%)	5 (100%)	*p* = 0.278
Atrial fibrillation (n, %)	11 (22%)	1 (20%)	*p* = 0.312
Laboratory data			
B-type natriuretic peptide (pg/ml)	606 (234–1240)	341 (77–657)	*p* = 0.303
Troponin T (ng/L)	66 (39–104)	134 (47–215)	*p* = 0.464
Echocardiography			
Left ventricular ejection fraction (%)	56 (43–63)	59 (51–60)	*p* = 0.823
Septal wall thickness (mm)	16 (13–18)	17 (13–20)	*p* = 0.578
Inferior wall thickness (mm)	15 (13–17)	15 (13–16)	*p* = 0.780
Left ventricular end diastolic diameter (mm)	42 (36–45)	41 (37–42)	*p* = 0.776
E/e’ (Average of lateral and septal e’)	20.6 (16–24)	18.1 (16.9–20.6)	*p* = 0.241
Lateral S′	5.5 (4–7)	6.25 (4,25–8.3)	*p* = 0.588

*AS* aortic valve stenosis; *wtTTR* wild-type transthyretin; *mTTR* mutant-type transthyretin; *NYHA* New York Heart Association. Values are presented as medians with interquartile ranges (IQR), or as percentages. The strength of the associations was calculated with the nonparametric Mann-Whitney test or the chi-square test, as appropriate

**Fig. 1:**
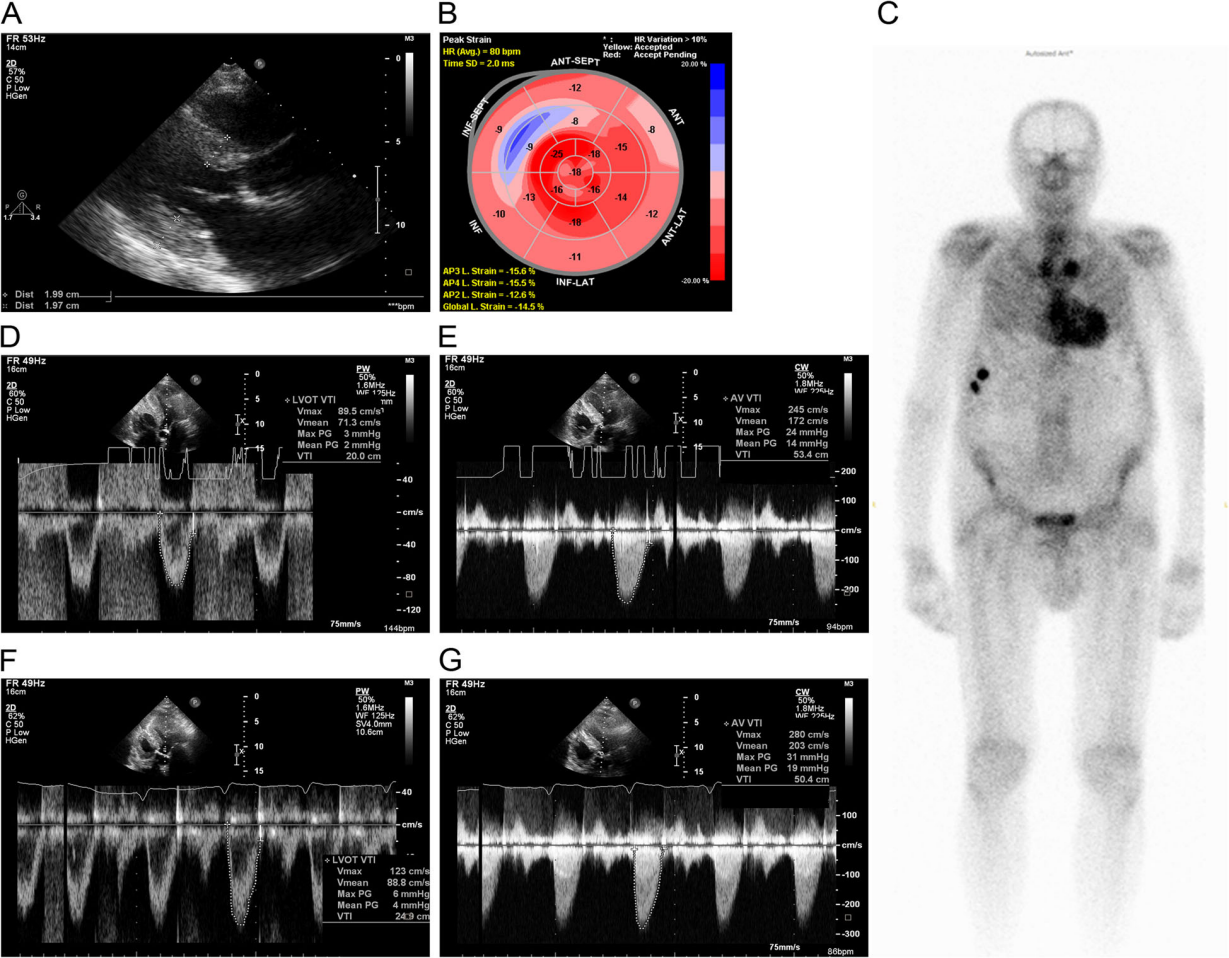
Images of a patient with wtTTR cardiac amyloidosis and low flow-low grade, pseudo-severe aortic valve stenosis. LVEF was 51%. **a**: Transthoracic echocardiography, parasternal long axis view. Septum and inferior wall are 20 mm thick at end-diastole. **b**: Bull’s eye image of the left ventricular longitudinal strain. Typical apical sparing. **c**: Pyrophosphate isotope scan, with significant take up of the tracer in the heart: Perugini score 3. **d-g**: PW and CW Doppler images of the left ventricular outflow tract at rest and at low dose dobutamine stress test. D: Resting PW Doppler, **e**: Resting CW Doppler. Resting calculated AVA: 0.54 cm2/BSA. **f**: PW Doppler at dobutamine test. **g**: CW Doppler at dobutamine test. Significant elevation in SV and AVA at dobutamine test. Calculated AVA at dobutamine test: 0.76 cm2/BSA

At baseline echo, an aortic valve area less than 0.6 cm^2^/body surface area (BSA) m^2^ was calculated in 3 cases, whereas in 2 cases, the calculated AVA was somewhat greater than 0.6 cm^2^/BSA (0.65 and 0.63 cm^2^/m^2^) – indicating moderate AS. In patients with an AVA/BSA less than 0.6 cm^2^, we performed dobutamine stress echo to distinguish between true-severe AS, and pseudo-severe AS. One patient, with a left ventricular ejection fraction (LVEF) of 48%, had true-severe AS: both echo and EKG suggested amyloidosis, CMR verified CA – and thus, the final diagnosis was AL amyloidosis (Table 2). This patient died within two months after diagnosis. In two patients, the stress test showed significant increase of AVA and thus provided evidence for pseudo-severe AS (Table 2) with a LVEF of 51 and 59%. One of these patients was an 89-year old male patient, originally diagnosed with colon cancer – and heart failure revealed by preoperative evaluation. Echo showed LFLG AS and severe left ventricular hypertrophy with relative low voltage on 12-lead ECG. The dobutamine stress echo proved pseudo-severe AS, and the PYP isotope scan was typical for TTR CA. Finally, hemicolectomy was performed without any major complication. The cardiac images of this patient are shown in Fig. 1. The other patient with AL CA and pseudo-severe LFLG AS was started on therapy for plasma cell dyscrasia.

**Table 2:** Characteristics of patients with CA and AS

Patient’s number, age (years) and sex	Type of CA	LV wall thickness (septum/posterior wall, mm) measured with echo, left ventricular ejection fraction (%), stroke volume index (ml/m2)	Presence of typical LGE in CMR or semiquantitative score > 1 at PYP isotope scan in TTR amyloid	Cardiac biopsy positive for amyloid	Apical sparing at strain analysis. (Average apical LS/(average basal LS + mid-LS))	AVA/BSA (cm2/m2), and mean aortic valve gradient at rest, measured by TTE echo	AVA/BSA (cm2/m2) and aortic valve gradient during dobutamine stress echo	Final diagnosis of the type of AS
1, 66, male	AL	17/16, 48, 20	yes	DNP	yes 0.75	0.45/BSA, 13	0.45/BSA, 22	True-severe AS
2, 68, female	AL	13/13, 60, 40	DNP	DNP	yes 0.77	0.65/14	DNP	Moderate AS
3, 89, male	wtTTR	20/20, 51, 22	yes	DNP	yes 0.81	0.54, 12	0.76, 19	Pseudo-severe AS
4, 69, female	AL	20/15, 61, 31	DNP	yes	yes 0.77	0.63, 22	DNP	Moderate AS
5, 83, female	AL	12/12, 59, 38	yes	DNP	yes 0.77	0.58, 19	0.86, 25	Pseudo-severe AS

*AVA* aortic valve area, *LS* longitudinal strain, *TTE* transthoracic echocardiography, *NA* not applicable, *DNP* did not performed. *CA* cardiac amyloidosis, *AS* aortic stenosis, *PYP* pyrophosphate, *TTR* transthyretin

Apical sparing, the difference of the LS between the apical and mid + basal segments of the left ventricle was obvious at visual assessment in bull’s eye images in all cases, but the ratio of average apical LS/(average basal LS + average mid LS) did not reach 1.

## Discussion

We found a 9% prevalence of moderate-to severe AS among consecutive, unselected CA patients. Of note, the vast majority of our CA patients had AL amyloidosis. This might be explained, in part, that our university hospital specializes also in hematology, in addition to cardiology. Nowadays, the number of patients diagnosed with wild-type TTR cardiac amyloidosis is growing fast [3]. However, our patients were diagnosed between 2009 and 2019, and this might be the other cause of the relatively small percentage of TTR patients.

In previous years, the prevalence of CA in AS patients was explored using different methods and study designs and was found to be 4.1 [9] to 16% [6]. However, all the publications on these studies assert that these patients had TTR, but not AL amyloidosis. In two studies, where the prognosis was analyzed in addition, the authors found a significantly worse prognosis for patients with both AS and CA than for those with AS only. This was true after valve replacement [9], as well as when pooled patients with and without aortic valve replacement were studied [7]. On the other hand, the prevalence of AS among CA patients was studied only by a few authors; however, they also used registries of TTR CA patients. Sperry et al. found that the prevalence of moderate to severe AS was 16% in TTR CA [11]. The retrospective analysis by Java et al. included patients with diverse causes of amyloidosis. They examined the results of aortic valve replacement in these patients [17]. The authors screened the database of Mayo Clinic (Rochester, MN, USA) for patients treated with aortic valve replacement and diagnosed with any type of amyloidosis in addition. Of the 16 patients meeting both criteria, only 6 had cardiac manifestations of amyloid disease; moreover, this subgroup of CA and AS patients was not analyzed separately, therefore this publication has a limited value for calculating the common prevalence of AS and CA. Based on these results, it appears that coexistence of TTR CA and AS is more common than that of AL CA and AS. A plausible explanation is the difference in the patients’ age. The prevalence of AS is strongly age dependent, just as wtTTR amyloidosis [2, 3] is, and thus the risk of coexistence is also increasing with age. The median age of patients with AL amyloidosis is lower, as PCD affects younger patients. Thus the risk of a coincidence with AS is less.

Cardiac amyloidosis was once thought to be a very rare disease [18, 19]. Studies in heart failure patients with preserved ejection fraction found CA to be surprisingly common, prevalence of CA was 17 and 29% in two different studies [20, 21]. An autopsy study found a 25% prevalence of wtTTR cardiac amyloidosis over the age of 85 years [22]. The above–mentioned, recent publications on CA and AS also suggest that CA is more common than previously thought. This new piece of information has therapeutic implications, as well as diagnostic importance. Our patient population is rather small and therefore, it is not possible to identify and describe echocardiographic, laboratory, clinical, and prognostic differences between these patients with CA or CA and AS. The novelty of our retrospective analysis is that we found moderate-to severe AS to be relatively common in a CA population, where AL amyloidosis was present in 80%.

The other novelty of our publication is, that it may draw attention to the use of dobutamine stress echo in LFLG AS patients with preserved ejection fraction in CA. Current guidelines do not recommend to perform dobutamine stress test with echo in LFLG AS patients with preserved ejection fraction (EF) [1]. The latest one by the European Society of Cardiology on the diagnosis and treatment of valvular heart disease [1] recognizes and underlines the diagnostic significance of LFLG AS. It recommends an integrative, stepwise diagnostic algorithm for these patients. First, the suspicion of AS should be raised by the morphology of the aortic valve. When the calculated aortic valve area (AVA) is less than 1 cm^2^, the mean pressure gradient is low (< 40 mmHg), and low-flow state is confirmed by the calculated low-stroke volume index (< 35 ml/m^2^), the guideline recommends performing dobutamine stress echocardiography. This test can differentiate between “pseudo-severe AS”, and true-severe AS. In pseudo-severe AS, AVA will increase together with stroke volume (SV), but the mean transvalvular gradient will not change significantly. In case of true-severe AS, the gradient will increase, but AVA will not change significantly. Of course, the dobutamine test will not aid the correct diagnosis in the absence of left ventricular contractile reserve – in this case, other clinical and imaging parameters should be taken into account. Interestingly, the guideline recommends performing the dobutamine test in LFLGAS only in patients with a reduced LVEF. From the pathophysiological perspective, this may seem reasonable, as one could speculate that only reduced LVEF can be increased by dobutamine. However, there are data on the usefulness and safety of this test in patients with LFLG AS, with reduced SV, but a normal EF. Caval et al [23] performed the dobutamine test in 55 patients with an average LVEF of 63% to differentiate true-severe AS from pseudo-severe AS. They concluded that the method is useful and safe in these patients. We also performed this test in three cases with LFLG AS (LVEF being 48, 51, and 58%), and it was diagnostic in all cases. In particular, the significant increase of AVA (Table 2) made it obvious that the symptoms of heart failure were caused by CA rather than pseudo-severe AS in two cases, and we found real, severe AS and CA in one case.

Although there are speculations that the pathomechanism of CA and AS may be connected, as amyloid deposition in the aortic valve could induce stenosis, there is no supporting evidence for that. Apparently, true-severe AS and CA coexist in a large number of patients. However, this can rather be explained simply by statistical distribution, as the prevalence of AS is quite high in the elderly, who also have a higher risk for wtTTR. In the above-mentioned studies, 6 to 16% of the AS patients systematically screened for TTR amyloidosis had wtTTR CA [6, 7, 9, 10]. These patients were either not screened for AL amyloidosis, or AL CA was also excluded. On the other hand, the incidence of AS is not mentioned in a report published earlier on a large series of CA patients. In a study of 149 wtTTR CA patients, where authors focused on the echocardiographic, ECG, and clinical characteristics, there was not even a single patient identified with any degree of AS [24]. This may raise concerns about the validity of these data, and also suggests that the possible coexistence of these two clinical entities should always be considered – this is necessary for establishing the correct diagnosis. We suppose that the simultaneous presence of pronounced left ventricular hypertrophy and diastolic dysfunction is a possible reason for overlooking CA in AS. In particular, these abnormalities are common in both disorders, and LVH could be interpreted as the consequence of AS. On the other hand, it is easy to overlook AS in CA, as gradients can be surprisingly low due to the low stroke volume often seen in CA [25]. Precise calculation of AVA – and when necessary, dobutamine stress echo – can clarify the diagnosis. Interestingly, we saw the phenomenon of “apical sparing” (preserved longitudinal endocardial strain in the apical versus basal segments) in all patients with CA and AS, which may be a useful prompt to look for CA when examining patients with AS. Of course, further “red flags” for CA should be evaluated, such as absolute or relative low voltage on the ECG, and other clinical signs of systemic amyloidosis.

In our study, only one AL patient had significant, true-severe AS. This 66-year old patient with advanced (NYHA Class III) heart failure survived only 2 months after diagnosis. In this case, life expectancy was clearly limited by AS, CA, and the systemic disease of multiple myeloma. In such a case, it is not possible to determine the share of AS and CA in contributing to heart failure. In a UK study [9], patients with TTR CA and severe AS had a lot worse postoperative prognosis than those with calcified AS: the mortality rate during the follow-up period was 50% (3/6) versus 7.5% (8/106). These data suggest that when CA and AS coexist, both diseases contribute to the symptoms, as well as to poor prognosis.

Sperry et al [11] at Cleveland Clinic Foundation (Ohio, USA) performed a retrospective analysis among 171 consecutive patients diagnosed with TTR CA at their institution. Diagnosis was based on endomyocardial biopsy, cardiac magnetic resonance, echocardiography (showing an apical sparing strain pattern), and/or 99 m-TcPYP scintigraphy. They compared the survival of patients with moderate (10 patients), low-flow, low-grade (11 patients), or high-grade (6 patients) AS to those without AS. Two-year mortality was high in both groups (37 and 33%), but did not differ statistically, although 11 AS patients underwent aortic valve repair. The authors concluded that routine screening for TTR cardiac amyloidosis in LFLG AS patients might be useful, as it may detect the disease and influence therapy. This study, however, did not include AL CA patients. The ratio of AS patients who had dobutamine echocardiography is not mentioned and therefore, the number of patients with true-severe or pseudo-severe AS is not known. The authors highlight the role of echocardiography with strain analysis and that of 99 m-TcPYP scintigraphy, but fail to mention the role of the dobutamine stress echo in the diagnostic process.

Based on our experience, we wish to underline the importance of dobutamine stress echocardiography in our LFLG AS population. This test can identify patients with true-severe AS, who may benefit from AVR or TAVI. We think that further stratification of LFLG AS, even in cases with preserved EF, is necessary in this patient population with CA, to screen for patients, who may benefit from invasive treatment.

## Conclusion

This is the first publication aimed to determine the prevalence of AS among unselected CA patients. In our patient population, the prevalence of moderate to severe AS was 9% based on resting echocardiography findings. Using dobutamine stress echocardiography, we were able to distinguish patients with pseudo-severe AS from those with true-severe AS, even in case of LFLGAS with preserved LVEF. Our results may draw attention to the coexistence of CA and AS, as well as to the importance of dobutamine echocardiography in determining the nature of AS in CA.

### Limitations

The major limitation of our study is its small sample size. Because hematology is a major component of the clinical profile of the 3rd Department of Internal Medicine at Semmelweis University, our study population is not representative of CA patients in Hungary – those with AL amyloidosis are possibly overrepresented. Nevertheless, according to our knowledge, this study is the first to screen consecutive CA patients for AS.

## Data Availability

The authors declare that all data supporting the findings of this study are available within the article.

## Abbreviations

AL amyloidosis: Light chain amyloidosis
AS: Aortic valve stenosis
AVA: Aortic valve area
BSA: Body surface area
CA: Cardiac amyloidosis
CMR: Cardiac magnetic resonance
EF: Ejection fraction
LFLG AS: Low flow-low grade aortic valve stenosis
LS: Longitudinal strain
LVEF: Left ventricular ejection fraction
LVH: Left ventricular hypertrophy
mTTR amyloidosis: Mutant type transthyretin amyloidosis
NYHA: New York Heart Association
PCD: Plasma cell dyscrasia
PYP: Pyrphoshate
TTR amyloidosis: Transthyretin amyloidosis
wtTTR: Wild-type transthyretin amyloidosis
